# Supplementary material for: Capturing the nature of events and event context using hierarchical event descriptors (HED)
Source: Neuroimage. Author manuscript; Available in PMC 2022 Mar 16. (PMC8925904; doi:10.1016/j.neuroimage.2021.118766)
Supplement: 1 [file NIHMS1770954-supplement-1.docx]

### **Supplementary Table 1:** Complete JSON event file for Wakeman-Henson dataset (short-form). The file has been re-spaced for readability. All of the definitions have been gathered into additional metadata dictionaries at the end of the file. We have included the text descriptions as BIDS Levels for categorical columns, but these could also be included in the HED annotations using the *Description* tag.

{

"onset": {

"Description": "Position of event marker in seconds relative to the start.",

"Units": "s"

},

"duration": {

"Description": "Duration of the event in seconds.",

"Units": "s"

},

"event_type": {

"LongName": "Event category",

"Description": "The main category of the event.",

"Levels": {

"show_face": "Display a face to mark end of pre-stimulus and start of blink-inhibition.",

"show_face_initial": "Display a face at the beginning of the recording.",

"show_circle": "Display a white circle to mark end of the stimulus and blink inhibition.",

"show_cross": "Display only a white cross to mark start of trial and fixation.",

"left_press": "Experiment participant presses a key with left index finger.",

"right_press": "Experiment participant presses a key with right index finger.",

"setup_left_sym": "Setup for experiment with pressing key with left index finger

means a face with above average symmetry.",

"setup_right_sym": "Setup for experiment with pressing key with right index finger

means a face with above average symmetry.",

"double_press": "Experiment participant presses both keys ."

},

"HED": {

"show_face": "Sensory-event, Experimental-stimulus, (Def/Face-image, Onset),

(Def/Blink-inhibition-task,Onset), (Def/Cross-only, Offset)",

"show_face_initial": "Sensory-event, Experimental-stimulus, (Def/Face-image, Onset),

(Def/Blink-inhibition-task, Onset), (Def/Fixation-task, Onset)",

"show_circle": "Sensory-event, (Intended-effect, Cue), (Def/Circle-only, Onset),

(Def/Face-image, Offset), (Def/Blink-inhibition-task, Offset), (Def/Fixation-task, Offset)",

"show_cross": "Sensory-event, (Intended-effect, Cue), (Def/Cross-only, Onset),

(Def/Fixation-task, Onset), (Def/Circle-only, Offset)",

"left_press": "Agent-action, Participant-response, Def/Press-left-finger",

"right_press": "Agent-action, Participant-response, Def/Press-right-finger",

"setup_left_sym": "Experiment-structure, (Def/Left-sym-cond, Onset),

(Def/Initialize-recording, Onset)",

"setup_right_sym": "Experiment-structure, (Def/Right-sym-cond, Onset),

(Def/Initialize-recording, Onset)",

"double_press": "Agent-action, Indeterminate-action, (Press, Keyboard-key)"

}

},

"face_type": {

"Description": "Factor indicating type of face image being displayed.",

"Levels": {

"famous_face": "A face that should be recognized by the participants.",

"unfamiliar_face": "A face that should not be recognized by the participants.",

"scrambled_face": "A scrambled face image generated by taking face 2D FFT."

},

"HED": {

"famous_face": "Def/Famous-face-cond",

"unfamiliar_face": "Def/Unfamiliar-face-cond",

"scrambled_face": "Def/Scrambled-face-cond"

}

},

"rep_status": {

"Description": "Factor indicating whether this image has been already seen.",

"Levels": {

"first_show": "Factor level indicating the first display of this face.",

"immediate_repeat": "Factor level indicating this face was the same as previous one.",

"delayed_repeat": "Factor level indicating face was seen 5 to 15 trials ago."

},

"HED": {

"first_show": "Def/First-show-cond",

"immediate_repeat": "Def/Immediate-repeat-cond",

"delayed_repeat": "Def/Delayed-repeat-cond"

}

},

"trial": {

"Description": "Indicates which trial this event belongs to.",

"HED": "Experimental-trial/#"

},

"rep_lag": {

"Description": "How face images before this one was the image was previously presented.",

"HED": "(Face, Item-interval/#)"

},

"stim_file": {

"Description": "Path of the stimulus file in the stimuli directory.",

"HED": "(Image, Pathname/#)"

},

"hed_def_sensory": {

"Description": "Metadata dictionary for gathering sensory definitions",

"HED": {

"cross_only_def": "(Definition/Cross-only, (Visual-presentation, (Foreground-view,

(White, Cross), (Center-of, Computer-screen)), (Background-view, Black),

Description/A white fixation cross on a black background in the center of the screen.))",

"face_image_def": "(Definition/Face-image, (Visual-presentation,

(Foreground-view, ((Image, Face, Hair), Color/Grayscale), ((White, Cross),

(Center-of, Computer-screen))), (Background-view, Black),

Description/A happy or neutral face in frontal or three-quarters frontal pose with long

hair cropped presented as an achromatic foreground image on a black background with

a white fixation cross superposed.))",

"circle_only_def": "(Definition/Circle-only, (Visual-presentation, (Foreground-view,

((White, Circle), (Center-of, Computer-screen))), (Background-view, Black),

Description/A white circle on a black background in the center of the screen.))"

}

},

"hed_def_actions": {

"Description": "Metadata dictionary for gathering participant action definitions",

"HED": {

"press_left_finger_def": "(Definition/Press-left-finger,

((Index-finger, (Left-side-of, Experiment-participant)), (Press, Keyboard-key),

Description/The participant presses a key with the left index finger to indicate

a face symmetry judgment.))",

"press_right_finger_def": "(Definition/Press-right-finger,

((Index-finger, (Right-side-of, Experiment-participant)), (Press, Keyboard-key),

Description/The participant presses a key with the right index finger to indicate

a face symmetry evaluation.))"

}

},

"hed_def_conds": {

"Description": "Metadata dictionary for gathering experimental condition definitions",

"HED": {

"famous_face_cond_def": "(Definition/Famous-face-cond, (Condition-variable/Face-type,

(Image, (Face, Famous)), Description/A face that should be recognized by the participants))",

"unfamiliar_face_cond_def": "(Definition/Unfamiliar-face-cond,

(Condition-variable/Face-type, (Image, (Face, Unfamiliar)),

Description/A face that should not be recognized by the participants.))",

"scrambled_face_cond_def": "(Definition/Scrambled-face-cond,

(Condition-variable/Face-type, (Image, (Face, Disordered)),

Description/A scrambled face image generated by taking face 2D FFT.))",

"first_show_cond_def": "(Definition/First-show-cond,

((Condition-variable/Repetition-type, (Item-count/1, Face), Item-interval/0),

Description/Factor level indicating the first display of this face.))",

"immediate_repeat_cond_def": "(Definition/Immediate-repeat-cond,

((Condition-variable/Repetition-type, (Item-count/2, Face), Item-interval/1),

Description/Factor level indicating this face was the same as previous one.))",

"delayed_repeat_cond_def": "(Definition/Delayed-repeat-cond,

(Condition-variable/Repetition-type, (Item-count/2, Face),

(Item-interval, (Greater-than-or-equal-to, Item-interval/5)),

Description/Factor level indicating face was seen 5 to 15 trials ago.))",

"left_sym_cond_def": "(Definition/Left-sym-cond, (Condition-variable/Key-assignment,

((Index-finger, (Left-side-of, Experiment-participant)), (Behavioral-evidence, Symmetrical)),

((Index-finger, (Right-side-of, Experiment-participant)), (Behavioral-evidence, Asymmetrical)),

Description/Left index finger key press indicates a face with above average symmetry.))",

"right_sym_cond_def": "(Definition/Right-sym-cond, (Condition-variable/Key-assignment,

((Index-finger, (Right-side-of, Experiment-participant)), (Behavioral-evidence, Symmetrical)),

((Index-finger, (Left-side-of, Experiment-participant)), (Behavioral-evidence, Asymmetrical)),

Description/Right index finger key press indicates a face with above average symmetry.))"

}

},

"hed_def_tasks": {

"Description": "Metadata dictionary for gathering task definitions",

"HED": {

"face_symmetry_evaluation_task_def": "(Definition/Face-symmetry-evaluation-task,

(Task, Experiment-participant, (See, Face), (Discriminate, (Face, Symmetrical)),

(Press, Keyboard-key),

Description/Evaluate degree of image symmetry and respond with key press evaluation.))",

"blink_inhibition_task_def": "(Definition/Blink-inhibition-task,

(Task, Experiment-participant, Inhibit-blinks,

Description/Do not blink while the face image is displayed.))",

"fixation_task_def": "(Definition/Fixation-task, (Task, Experiment-participant, (Fixate, Cross),

Description/Fixate on the cross at the screen center.))"

}

},

"hed_def_setup": {

"Description": "Metadata dictionary for gathering setup definitions",

"HED": {

"setup_def": "(Definition/Initialize-recording, (Recording))"

}

},

"value": {

"Description": "Numerical event marker",

"Levels": {

"x0": "Disappearance of face image and display of the inter-stimulus circle simultaneously",

"x1": "Disappearance of face image and display of the inter-stimulus circle simultaneously",

"x2": "Initial setup with left finger key press indicating above average symmetry",

"x3": "Initial setup with right finger key press indicating above average symmetry",

"x5": "Initial presentation of famous face",

"x6": "Immediate repeated presentation of famous face",

"x7": "Delayed repeated presentation of famous face",

"x13": "Initial presentation of unfamiliar face",

"x14": "Immediate repeated presentation of unfamiliar face",

"x15": "Delayed repeated presentation of unfamiliar face",

"x17": "Initial presentation of scrambled face",

"x18": "Immediate repeated presentation of scrambled face",

"x19": "Delayed repeated presentation of scrambled face",

"x256": "Left finger key press",

"x4096": "Right finger key press",

"x4352": "Left and right finger key presses"

}

}

}

### 
