# Supplementary material for: Capturing the nature of events and event context using hierarchical event descriptors (HED)
Source: Neuroimage. Author manuscript; Available in PMC 2022 Mar 16. (PMC8925904; doi:10.1016/j.neuroimage.2021.118766)
Supplement: 2 [file NIHMS1770954-supplement-2.docx]

##### Supplementary Table 2: The assembled form of the HED annotation for the second event in Table 3 (as shown in Table 5) and in three different forms expanded by tools. Form 1 is the form that would normally appear in the ...events.json sidecar and be viewed. The tag strings have been re-spaced and partially bolded for readability.

##### Form 1: Short-form annotation of the sensory event corresponding to the first showing of famous face image f032.bmp. Definitions are unexpanded (as shown in Table 7).

| *Sensory-event, Experimental-stimulus, (Def/Face-image, Onset), (Def/Blink-inhibition-task, Onset), (Def/Cross-only, Offset), Def/Famous-face-cond, Def/Immediate-repeat-cond,*  *(Item-interval/1),(Image, Pathname/f032.bmp)* |
| --- |

##### Form 2: Long-form annotation of the sensory event corresponding to an immediate reshowing of famous face image f032.bmp. Definitions are unexpanded. Terms from Form 1 are shown in bold.

| *Event/****Sensory-event****,*  *Property/Task-property/Task-event-role/****Experimental-stimulus****, (Property/Organizational-property/****Def/Face-image****,*  *Property/Data-property/Data-marker/Temporal-marker/****Onset****),*  *(Property/Organizational-property/****Def/Blink-inhibition-task****,*  *Property/Data-property/Data-marker/Temporal-marker/****Onset****),*  *(Property/Organizational-property/****Def/Cross-only****,*  *Property/Data-property/Data-marker/Temporal-marker/****Offset****),*  *Property/Organizational-property/****Def/Famous-face-cond****,*  *Property/Organizational-property/****Def/Immediate-repeat-cond****,*  *(Property/Data-property/Data-value/Quantitative-value/****Item-interval/1****)*  *(Item/Object/Man-made-object/Media/Visualization/****Image****,*  *Property/Informational-property/Metadata/****Pathname/f032.bmp****)* |
| --- |

#####

##### Form 3: Short-form annotation of the sensory event corresponding to the immediate reshowing famous face image f032.bmp. Definitions are expanded. The annotation has been manually indented to improve readability.

| ***Sensory-event****,*  ***Experimental-stimulus****,*  *((Def-expand/****Face-image****,*  *(Visual-presentation, (Foreground-view, ((Image, Face, Hair), Color/Grayscale),*  *((White, Cross), (Center-of, Computer-screen))), (Background-view, Black),*  *Description/A happy or neutral face in frontal or three-quarters frontal pose with long hair cropped*  *presented as an achromatic foreground image on a black background with a white fixation cross*  *superposed.)),* ***Onset****),*  *((Def-expand/****Blink-inhibition-task****,*  *(Task, Experiment-participant, Inhibit-blinks,*  *Description/Do not blink while the face image is displayed.)),* ***Onset****),*  *((Def-expand/****Cross-only****,*  *(Visual-presentation, (Foreground-view, (White, Cross), (Center-of, Computer-screen)),*  *(Background-view, Black),*  *Description/A white fixation cross on a black background in the center of the screen.)),* ***Offset****),*  *(Def-expand/****Famous-face-cond****, (Condition-variable/Face-type,*  *(Image, (Face, Famous)), Description/A face that should be recognized by the participants)),*  *(Def-expand/****Immediate-repeat-cond****, (Condition-variable/Repetition-type, (Item-count/2, Face),*  *Description/Factor level indicating the first display of this face.)),*  *Item-interval/1,*  *(****Image****,* ***Pathname/f032.bmp****)* |
| --- |

**Form 4:** Long-form annotation of the sensory event corresponding to the first showing of famous face image f032.bmp. Definitions are expanded.

| *Event/****Sensory-event****,*  *Property/Task-property/Task-event-role/****Experimental-stimulus****,*  *((Property/Organizational-property/****Def-expand/Face-image****,*  *(Property/Sensory-property/Sensory-presentation/Visual-presentation,*  *(Property/Sensory-property/Sensory-presentation/Visual-presentation/Foreground-view,*  *((Item/Object/Man-made-object/Media/Visualization/Image,*  *Item/Biological-item/Anatomical-item/Body-part/Head/Face,*  *Item/Biological-item/Anatomical-item/Body-part/Head/Hair),*  *Property/Sensory-property/Sensory-attribute/Visual-attribute/Color/Grayscale),*  *((Property/Sensory-property/Sensory-attribute/Visual-attribute/Color/CSS-color/White-color/White,*  *Item/Object/Geometric-object/2D-shape/Cross),*  *(Relation/Spatial-relation/Center-of,*  *Item/Object/Man-made-object/Device/IO-device/Output-device/Display-device/Computer-screen))),*  *(Property/Sensory-property/Sensory-presentation/Visual-presentation/Background-view,*  *Property/Sensory-property/Sensory-attribute/Visual-attribute/Color/CSS-color/Gray-color/Black),*  *Property/Informational-property/Description/A happy or neutral face in frontal or three-quarters frontal pose with long hair cropped presented as an achromatic foreground image on a black background with a white fixation cross superposed.)),*  *Property/Data-property/Data-marker/Temporal-marker/****Onset****),*  *((Property/Organizational-property/****Def-expand/Blink-inhibition-task****,*  *(Property/Organizational-property/Task,*  *Property/Agent-property/Agent-task-role/Experiment-participant,*  *Action/Move/Move-body-part/Move-eyes/Inhibit-blinks,*  *Property/Informational-property/Description/Do not blink while the face image is displayed.)),*  *Property/Data-property/Data-marker/Temporal-marker/****Onset****)*  *((Property/Organizational-property/****Def-expand/Cross-only****,*  *(Property/Sensory-property/Sensory-presentation/Visual-presentation,*  *(Property/Sensory-property/Sensory-presentation/Visual-presentation/Foreground-view,*  *(Property/Sensory-property/Sensory-attribute/Visual-attribute/Color/CSS-color/White-color/White,*  *Item/Object/Geometric-object/2D-shape/Cross),*  *(Relation/Spatial-relation/Center-of,*  *Item/Object/Man-made-object/Device/IO-device/Output-device/Display-device/Computer-screen)),*  *(Property/Sensory-property/Sensory-presentation/Visual-presentation/Background-view,*  *Property/Sensory-property/Sensory-attribute/Visual-attribute/Color/CSS-color/Gray-color/Black),*  *Property/Informational-property/Description/A white fixation cross on a black background in the center of the screen.)),*  *Property/Data-property/Data-marker/Temporal-marker/****Offset****),*  *(Property/Organizational-property/****Def-expand/Famous-face-cond****,*  *(Property/Organizational-property/****Condition-variable/Face-type****,*  *(Item/Object/Man-made-object/Media/Visualization/Image,*  *(Item/Biological-item/Anatomical-item/Body-part/Head/Face,*  *Property/Data-property/Data-value/Categorical-value/Categorical-judgment-value/Famous)),*  *Property/Informational-property/Description/A face that should be recognized by the participants)),*  *(Property/Organizational-property/****Def-expand/Immediate-repeat-cond****,*  *(Property/Organizational-property/****Condition-variable/Repetition-type****,*  *(Property/Data-property/Data-value/Quantitative-value/Item-count/2,*  *Item/Biological-item/Anatomical-item/Body-part/Head/Face),*  *Property/Informational-property/Description/Factor level indicating the first display of this face.)),*  *Property/Data-property/Data-value/Quantitative-value/Item-interval/1,*  *(Item/Object/Man-made-object/Media/Visualization/Image,*  *Property/Informational-property/Metadata/Pathname/f032.bmp)* |
| --- |
